# Caffeine and Placebo Improved Maximal Exercise Performance Despite Unchanged Motor Cortex Activation and Greater Prefrontal Cortex Deoxygenation

**DOI:** 10.3389/fphys.2018.01144

**Published:** 2018-08-17

**Authors:** Flavio O. Pires, Carlos A. S. dos Anjos, Roberto J. M. Covolan, Eduardo B. Fontes, Timothy D. Noakes, Alan St Clair Gibson, Fernando H. Magalhães, Carlos Ugrinowitsch

**Affiliations:** ^1^Exercise Psychophysiology Research Group, School of Arts, Sciences and Humanities, University of São Paulo, São Paulo, Brazil; ^2^Neurophysics Group, Gleb Wataghin Physics Institute, University of Campinas, Campinas, Brazil; ^3^Research Group in Physical Activity, Cognition and Behavior, Federal University of Rio Grande do Norte, Natal, Brazil; ^4^Sports Science Institute of South Africa, Department of Human Biology, University of Cape Town, Cape Town, South Africa; ^5^Faculty of Health and Science, University of Essex, Colchester, United Kingdom; ^6^School of Physical Education and Sport, University of São Paulo, São Paulo, Brazil

**Keywords:** brain regulation, prefrontal cortex, performance, fatigue, VO_2MAX_

## Abstract

Caffeine (CAF) is an ergogenic aid used to improve exercise performance. Independent studies have suggested that caffeine may have the ability to increase corticospinal excitability, thereby decreasing the motor cortex activation required to generate a similar motor output. However, CAF has also been suggested to induce a prefrontal cortex (PFC) deoxygenation. Others have suggested that placebo (PLA) may trigger comparable effects to CAF, as independent studies found PLA effects on motor performance, corticospinal excitability, and PFC oxygenation. Thus, we investigated if CAF and CAF-perceived PLA may improve motor performance, despite the likely unchanged MC activation and greater PFC deoxygenation. Nine participants (26.4 ± 4.8 years old, VO_2MAX_ of 42.2 ± 4.6 mL kg^-1^ min^-1^) performed three maximal incremental tests (MITs) in control (no supplementation) and ∼60 min after CAF and PLA ingestion. PFC oxygenation (near-infrared spectroscopy at Fp1 position), MC activation (EEG at Cz position) and vastus lateralis and rectus femoris muscle activity (EMG) were measured throughout the tests. Compared to control, CAF and PLA increased rectus femoris muscle EMG (*P* = 0.030; *F* = 2.88; *d* = 0.84) at 100% of the MIT, and enhanced the peak power output (*P* = 0.006; *F* = 12.97; *d* = 1.8) and time to exhaustion (*P* = 0.007; *F* = 12.97; *d* = 1.8). In contrast, CAF and PLA did not change MC activation, but increased the PFC deoxygenation as indicated by the lower O_2_Hb (*P* = 0.001; *F* = 4.68; *d* = 1.08) and THb concentrations (*P* = 0.01; *F* = 1.96; *d* = 0.7) at 80 and 100% the MIT duration. These results showed that CAF and CAF-perceived PLA had the ability to improve motor performance, despite unchanged MC activation and greater PFC deoxygenation. The effectiveness of CAF as ergogenic aid to improve MIT performance was challenged.

## Introduction

Caffeine (CAF) is one of the most widely used ergogenic aid to improve exercise performance, although its underlying mechanisms of action are not completely understood. While skeletal muscle effects cannot be ruled out (Kalmar and Cafarelli, 1999), the most convincing mechanism is the CAF effect on neuronal A_1_ adenosine receptors in the central nervous system (Kalmar, 2005). Accordingly, CAF has the ability to increase neuronal activity and excitability at the spinal and supra-spinal levels, thereby improving the motor performance (Walton et al., 2002; Kalmar, 2005). For example, a previous study observed that CAF decreased the primary motor cortex (MC) activation required to generate a similar muscle activation and motor output during isometric knee extensions when compared to a control condition (de Morree et al., 2014). However, it is interesting to note that the antagonistic effect of CAF on A_1_ and A_2A_ adenosine receptors may lead to cerebral vasoconstriction together with an increased neuronal excitability (Yang et al., 2015), as CAF ingestion may reduce cerebral perfusion (Nehlig et al., 1992) and prefrontal cortex (PFC) oxygenation (Yang et al., 2015). These results may be paradoxical because PFC oxygenation has been suggested to play a role on the exercise tolerance when regulating the motor output (Ridderinkhof et al., 2004; Robertson and Marino, 2015a; Loehrer et al., 2016). Therefore, independent results suggest that CAF has the potential to improve motor performance, despite reducing MC activation and PFC oxygenation (Xu et al., 2015). Studies investigating MC activation and motor output simultaneously to PFC deoxygenation during CAF-supplemented exercises are required to better elucidate these complex responses to CAF ingestion.

Furthermore, some have argued that CAF effects on motor performance may be due to a placebo (PLA) effect (Foad et al., 2008). In fact, a recent study observed that participants perceiving PLA as CAF improved motor performance during a maximal incremental test (MIT), regardless of physiological alterations (Brietzke et al., 2017). Although the mechanisms underlying the PLA effect have not been fully described, PLA may trigger similar cerebral effects to CAF ingestion. For example, studies have reported a cognitive top–down effect on corticospinal excitability following different PLA settings, suggesting a decreased cortical activation necessary to generate the same motor output (Pollo et al., 2008; Fiorio et al., 2014). Moreover, a study observed changes in PFC oxygenation during a PLA-deceived intervention (Tian et al., 2009), thereby suggesting an expectation-induced cerebral response in the expected direction of the active substance (Wager et al., 2004, 2011; Liu, 2017). Hence, some of the effects of CAF on motor performance and cerebral responses to exercise may be related to a PLA effect. Unfortunately, no study has investigated the effects of ingesting CAF-perceived PLA on motor performance, MC activation and PFC deoxygenation.

The use of double-blind design approaches in CAF interventions have been challenged as the expectancy of ingesting CAF, rather than its isolated pharmacological effects, may influence exercise performance outcomes (Kirsch and Weixel, 1988; Dömötör et al., 2015). Since the use of a CAF-perceived PLA design is rare in the exercise literature (Foad et al., 2008; Brietzke et al., 2017), a design study including CAF, PLA perceived as CAF and control condition could help elucidate the actual PLA effect on motor performance and cerebral responses to exercise. Therefore, the present study was designed to investigate if CAF and PLA perceived as CAF may improve motor performance during MIT, regardless of a likely unchanged MC activation and increased PFC deoxygenation. We used a MIT paradigm to investigate the effects of CAF and CAF-perceived PLA on exercise performance and cerebral responses as the exercise-induced hypocapnia from 80% of the peak power output (W_PEAK_) may be insightful to evidence an increase in motor performance, regardless of MC and PFC changes. Thus, we hypothesized that compared to a control condition (i.e., no supplementation) CAF and CAF-perceived PLA would lead to a greater motor output (i.e., ↑ MIT performance) despite unchanged MC activation and higher PFC deoxygenation.

## Materials and Methods

### Participants

Nine healthy and physically active males volunteered to take part in this study (26.4 ± 4.8 years old, weight of 77.6 ± 12.1 kg, height of 171.7 ± 6.9 cm, and VO_2MAX_ of 42.2 ± 4.6 mL kg^-1^ min^-1^). They were non-smokers and free from neuromuscular or cardiopulmonary disorders. Some of these participants (*n* = 6) consumed caffeine as a coffee drink, having some (*n* = 3) consuming less than 120 mg of caffeine per day and others (*n* = 3), less than 44.5 mg of caffeine per day. The remaining participants (*n* = 3) did not habitually consume caffeine. We explained all risks and benefits of the experimental procedures before participants have signed an informed consent form; a local Ethics Committee (Protocol No. 0023.0.342.000-10) previously approved this study.

### Experimental Design

The experimental design consisted of (1) a familiarization with procedures and a preliminary MIT; (2) a control MIT (baseline); (3) a MIT after caffeine ingestion (CAF); (4) a MIT after placebo (PLA) perceived as caffeine ingestion. After performing session 1, participants performed sessions 2, 3, and 4 in a randomized, counterbalanced order, at the same time of day in a laboratory environment (≈21°C temperature and ≈60% relative humidity). A 3–7 days washout period was used between sessions, to eliminate residual effects of fatigue and substance ingestion. Participants were encouraged to abstain from intense exercise and ingestion of caffeine, alcohol, and stimulant beverages for the 24 h before the sessions, as well as to maintain their daily physical activity routines throughout the period of the study. In order to control carbohydrate ingestion effects on CAF supplementation, participants were strongly recommended to maintaining their habitual diet (∼60% CHO, ∼20% protein, and ∼20% fat) while they were committed to the study.

Measures of PFC oxygenation, MC activation, and muscle activity were obtained throughout each MIT using near-infrared spectroscopy (NIRS), EEG and electromyography (EMG) techniques, respectively. Additionally, we measured oxygen uptake (VO_2_) in a breath-by-breath frequency, as previously reported in a companion paper (Brietzke et al., 2017).

### Maximal Incremental Tests

Instead of using a conventional laboratory cycle ergometer, all the MITs were performed on a road bicycle (Giant^®^, United States) attached to a cycle-simulator (Racer Mate, Computrainer, Seattle, WA, United States), and adapted with comfortable saddle and pedals for non-cyclists, as participants used their bicycles as preferential transport mode by the time the study was conducted. Participants arrived at the laboratory and were prepared for NIRS, EEG, and EMG data collection (as detailed ahead). Following an initial period of accommodation to the bicycle, they closed the eyes and remained quiet for 2 min for baseline measurements. Thereafter, they performed a standard warm-up consisting of a 5 min self-paced exercise (free gear and pedal cadence) and a 2 min controlled-paced exercise (100 W at 80 rpm). When participants were still cycling at 100 W, the MIT started with 25 W increments every minute until voluntary exhaustion, which was determined when the pedal cadence dropped below 80 rpm, in spite of three strong verbal encouragements. The cycle-simulator was calibrated before each test according to manufacturer’s instructions. Validity and reliability of the cycle-simulator power output values have been reported elsewhere (Peveler, 2013).

### Caffeine Supplementation and Placebo

Caffeine and placebo perceived as caffeine were ingested 1 h before the commencement of the MIT exercises. The substances were manipulated as 6 mg kg^-1^ of body mass of caffeine or placebo (sucrose) and offered in opaque capsules of equal size, color, and taste, to blind participants to treatments. Participants were told the study was investigating the reproducibility of CAF as a potential supplementation to improve exercise performance, thus they ingested PLA perceiving it as CAF. We approached the placebo intervention with a placebo-deceived design as others have previously argued that the use of double-blind designs is a possible source of bias in clinical trials and sports nutrition studies (Kirsch and Weixel, 1988; Saunders et al., 2016). In fact, some have reported that performance outcomes in physical tests are negatively affected if participants identify the presence of a PLA intervention (Beedie et al., 2006; Foad et al., 2008). Therefore, similar to designs reported elsewhere we informed participants of the presence of a PLA substance only when they concluded the participation in the study (Beedie et al., 2006; Foad et al., 2008). An investigator, unaware of the supplement given to participants, provided verbal encouragement during all MIT exercises.

### Motor Output and Muscle Activation

The peak power output (W_PEAK_), determined as the highest power output attained in the test, indicated the MIT motor output. The absolute time to exhaustion was further used as a performance indicator.

Activation of the vastus lateralis (VL) and rectus femoris (RF) muscles was monitored through an EMG unit (Delsys, Chicago, IL, United States). A pair of EMG electrodes (an inter-electrode distance of 2 cm) was placed over the belly of the VL and RF muscles following the probable muscle fiber orientation. Before electrode placement, the skin was shaved, exfoliated, and cleaned with isopropyl alcohol. Then, the position of the electrodes on the skin was marked with a surgical pen to ensure the same electrode placement between testing sessions.

The EMG signal was amplified (gain 1,000) and sampled at 2 kHz with a hardware band-pass filter set at 20 and 500 Hz. After baseline subtraction, the root-mean-square value (RMS) of the EMG signal obtained over the controlled-pace warm-up (i.e., 100 W and 80 rpm) was used to normalize EMG values obtained during MIT exercises. Thereafter, the RMS of VL and RF muscles was averaged over the last 10 s of every 20% of the total exercise duration.

### Prefrontal Cortex Oxygenation

The PFC oxygenation level was assessed through changes in oxy- (O_2_Hb) and deoxy-hemoglobin concentrations (HHb) by using a continuous-wave NIRS (CW6- TechEn, Inc., Milford, MA, United States), from the baseline period to the end of the MITs. This technology monitors the tissue absorption with optical fiber optodes, light sources, and detectors. The micromolar (μM) changes in O_2_Hb and HHb concentrations over time were obtained at a 25 Hz frequency, using a modified Beer-Lambert law calculation with optical densities from continuous wavelengths of 690 nm (differential pathlength factor of 6.15) and 830 nm (differential pathlength factor of 4.77) (Duncan et al., 1995). The validity and reliability of NIRS technology have been reported and reviewed (Van Beekvelt et al., 2001; Ekkekakis, 2009). The optodes were placed over the prefrontal lobe at the Fp1 position (an inter-optode distance of 4.5 cm), according to the international EEG 10–20 system. A plastic holder housed the optodes, an adhesive tape was used to maintain them at the Fp1 position.

Raw NIRS data were initially filtered with a 0.4 Hz low pass-band filter (HomER^1^) and then resampled to 1 Hz. Thereafter, the exercise NIRS data were normalized by values recorded in the least 30 s of the baseline period (Δμmol), when the participants were completely calm with eyes closed. Briefly, we used this post-absorptive period for normalization purposes as the use of a pre-absorptive baseline period ∼45–60 min before the exercise could have increased the random error in cerebral measures, as participants would be allowed to move around during this interval (using toilets, sitting down, or standing up, etc.). This would increase the likelihood of NIRS optodes and EEG electrodes displacement due to sweat and friction with the skin, as observed in a pilot study. Moreover, a pre-absorptive baseline normalization would not represent an interference-free period, as the expectation of receiving a given treatment during placebo manipulation (i.e., deceptive manipulation) seems to produce cerebral responses in the expected direction of the active substance (Wager et al., 2004, 2011; Tian et al., 2009; Liu, 2017). Thus, O_2_Hb, HHb, and total hemoglobin concentration (THb = O_2_Hb + HHb) responses over the last 10 s of every 20% of the total test duration were used to indicate PFC oxygenation and blood volume, respectively.

Importantly, we used relative rather than absolute intensities to verify CAF and PLA effects on cerebral responses to exercise as we expected that these substances would affect the PFC oxygenation, mainly under exercise-induced hypocapnia from 80% W_PEAK_. As CAF ingestion also improves performance, the analysis of relative intensities is appropriate to prevent the comparison of equivalent absolute power output values (e.g., 200 W) that differed in %W_PEAK_ and hyperventilation-induced hypocapnia effects. However, to estimate the actual CAF and CAF-perceived PLA effects on cerebral vasoconstriction and oxygenation we further compared NIRS responses during cycling at 150 W. We used this mild exercise intensity to exclude metabolic-related confounding factors when comparing NIRS responses to exercise.

### Primary Motor Cortex Activation

The MC activation was obtained with an EEG unit (NicoletOne V32, Viasys Healthcare, Inc., Madison, WI, United States). Ag–AgCl electrodes (resistance < 5 kΩ) were placed on the scalp after exfoliation and cleaning, adhesive tape was used to fix the electrodes at the Cz position (according to the international EEG 10–20 system). This position was chosen because this area is known to generate the motor drive to the lower limb muscles, according to a motor homunculus model (Goodall et al., 2012). The surface signal was amplified (gain of 1,000) and sampled at 2 kHz, thereafter the signal was resampled at 128 Hz. This procedure is in accordance with that used by others to assess cortical activation during exercise (Robertson and Marino, 2015b).

The EEG data recorded during exercise were normalized by values within the last 30 s of the baseline period. Data in frequency domain were used to calculate the EEG power spectrum density through the Welch periodogram of detrended data, with a 0.2 Hz resolution. As a result, the area under the power spectrum curve between 8 and 13 Hz (alpha wave) was calculated for the last 10 s of every 20% of exercise. Importantly, the alpha bandwidth (α) was used to indicate MC activation as alpha wave reflects an increased number of neurons coherently activated (Pfurtscheller and Lopes da Silva, 1999). In this regard, increases in the inhibited neurons-to-disinhibited neurons relationship in MC may suggest a higher cortical activation, being this response likely associated with a facilitation of sensory stimuli derived from PFC areas (Uusberg et al., 2013). Consequently, an increased alpha bandwidth during exercise could indicate a cooperative-synchronized behavior of a large number of activated neurons, therefore suggesting a cortical facilitation (von Stein and Sarnthein, 2000; Robertson and Marino, 2015b).

### Gaseous Exchange Measures

Continuous gaseous exchange responses were measured with an open-system gas analyzer (Cosmed, Quark PT, Albano Laziale, Rome, Italy) from the baseline to the end of the MIT. The gas analyzer was calibrated according to manufacturer’s recommendation, using a 3 L syringe (Quinton Instruments, Milwaukee, WI, United States) before each test. The participants wore a mask (Hans Rudolph, Lenexa, KS, United States) connected to the gas analyzer for breath-by-breath measurements of VO_2_, ventilation volume (VE) and end-expired fraction CO_2_ (FeCO_2_). After data collection, breath-by-breath data were filtered using moving averages so that values greater than the third standard deviation (SD) from the local mean (the 5-breath moving average) were substituted by the local mean (Myers et al., 1990). Thereafter, a cubic spline interpolation technique was used to provide data at 1 Hz (DiMenna et al., 2008). Responses of VO_2_ (mL⋅kg^-1^⋅min^-1^), VE (L⋅min^-1^) and FeCO_2_ (%) within the last 10 s of every 20% of the test duration were averaged.

### Statistical Analyses

The sample size was previously calculated, considering a power ≥ 0.80 and a very large effect size (ES) as expressed as Cohen’s *d* (i.e., ≥0.6), according to suggestion described elsewhere (Hopkins et al., 2009). Thus, assuming a repeated-measures within-between interaction design (four repeated measures and three interventions), a sample size with 9 volunteers would be required (G^∗^Power 3 software). We further estimated a ∼20% dropout so that we initially included 11 participants in the study. Two individuals dropped out because of personal reasons (27.5 ± 0.7 years old, W_PEAK_ 255.3 ± 7.4 W and VO_2MAX_ of 42.2 ± 0.7 mL kg^-1^ min^-1^), therefore the final sample size was composed of nine participants.

We compared VO_2MAX_ and motor output responses such as W_PEAK_ and time to exhaustion in control, CAF and PLA conditions through a repeated-design mixed model, having ingestion (i.e., control, CAF or PLA ingestion) as a fixed factor and participants as a random factor. In case of significant *F*-values, multiple comparisons were corrected by Bonferroni’s test. Moreover, a number of mixed models, having time and ingestion as fixed factors, and participants as a random factor, was used to compare cerebral responses (NIRS and EEG measures), muscle activation (VL and RF RMS measures) and cardiopulmonary (VO_2_ and VE) responses over MIT exercises. Hence, dependent variables in these comparisons were O_2_Hb, HHb, and THb concentrations, EEG α bandwidth, VL and RF RMS as well as VO_2_, VE, and FeCO_2_ measures. Bonferroni’s adjustment was used for multiple comparison purposes. Significant results were accepted if *P* ≤ 0.05. Importantly, we performed a *post hoc* power analysis and all significant results elicited a power ≥ 0.80. Additionally, ES was also calculated afterward, thereby making easier comparisons with the literature. We calculated ES as η^2^, but thereafter converted and expressed as Cohen’s *d*, and interpreted as small (≤0.1), moderate (0.1 > and < 0.3), large (0.3 > and < 0.5), very large (0.5 > and < 0.9), and extremely large (≥0.9) (Hopkins et al., 2009). The mean and standard deviation (± SD) were used to express the results.

## Results

### Muscle Activation and Motor Output

**FIGURE 1:**
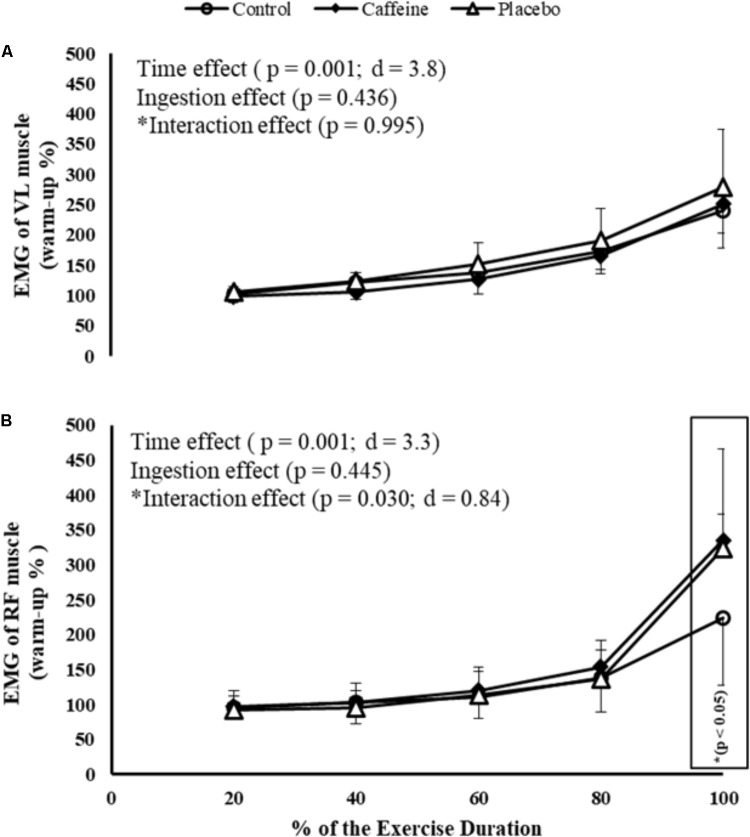
EMG of vastus lateralis (VL; **A**) and rectus femoris (RF; **B**) muscles during maximal incremental test (MIT) in control, caffeine and placebo (perceived as caffeine). Main effects of time and ingestion and time by ingestion interaction effects were reported in the figure. Values are mean (± SD).

Regarding EMG responses, there was a main time effect in VL (*P* = 0.0001; *F* = 58.22; *d* = 3.8) and RF (*P* = 0.0001; *F* = 43.47; *d* = 3.3) muscles. Despite no ingestion main effect (*P* = 0.445), an ingestion by time interaction effect was observed in RF (*P* = 0.030; *F* = 2.88; *d* = 0.84), thus participants elicited greater RF RMS at 100% of the MIT duration in CAF (*P* = 0.012) and PLA (*P* = 0.022) sessions when compared to control session. Nevertheless, neither ingestion (*P* = 0.436) nor ingestion by time interaction effect (*P* = 0.945) was observed in VL muscle (**Figures 1A,B**).

Furthermore, there was a significant ingestion main effect in W_PEAK_ (*P* = 0.006; *F* = 12.97; *d* = 1.8) and time to exhaustion (*P* = 0.007; *F* = 12.97; *d* = 1.8). Performance in CAF (272.1 ± 38.4 W, *P* = 0.016; 472 ± 90 s, *P* = 0.017) and PLA (273.2 ± 34.6 W, *P* = 0.010; 480 ± 80 s, *P* = 0.011) was higher than in control (244.7 ± 29.5 W and 409 ± 65 s) so that MIT after CAF and CAF-perceived PLA was greater than control when expressed as W_PEAK_ (11.2 and 11.9%, respectively) as well as time to exhaustion (15.4 and 17.4%, respectively).

### Cerebral Responses

When analyzing brain oxygenation responses to exercise performed at 150 W, it was found an ingestion main effect (*P* = 0.003; *F* = 9.22; *d* = 1.52) as both CAF (3.0 ± 5.0 Δμmol/L; *P* = 0.003) and PLA (5.0 ± 7.0 Δμmol/L; *P* = 0.023) reduced O_2_Hb concentrations when compared to control (11.0 ± 7.0 Δμmol/L). This reduction with CAF and PLA ingestions represented a decrease of 87.5 and 75.5% in relation to control, respectively. Additionally, there was an ingestion main effect on HHb concentrations (*P* = 0.025; *F* = 4.44; *d* = 1.053) as CAF (1.9 ± 3.5 Δμmol/L; *P* = 0.024), but not PLA (1.3 ± 3.4 Δμmol/L; *P* = 0.373), increased HHb concentrations when compared to control (-0.5 ± 2.2 Δμmol/L). In addition, there was an ingestion main effect on THb concentrations (*P* = 0.066; *F* = 3.15; *d* = 0.88) as CAF (5.0 ± 6.0 Δμmol/L; *P* = 0.070), but not PLA (7.0 ± 7.0 Δμmol/L; *P* = 0.254) reduced THb concentrations when compared to control (11.0 ± 5.0 Δμmol/L).

**FIGURE 2:**
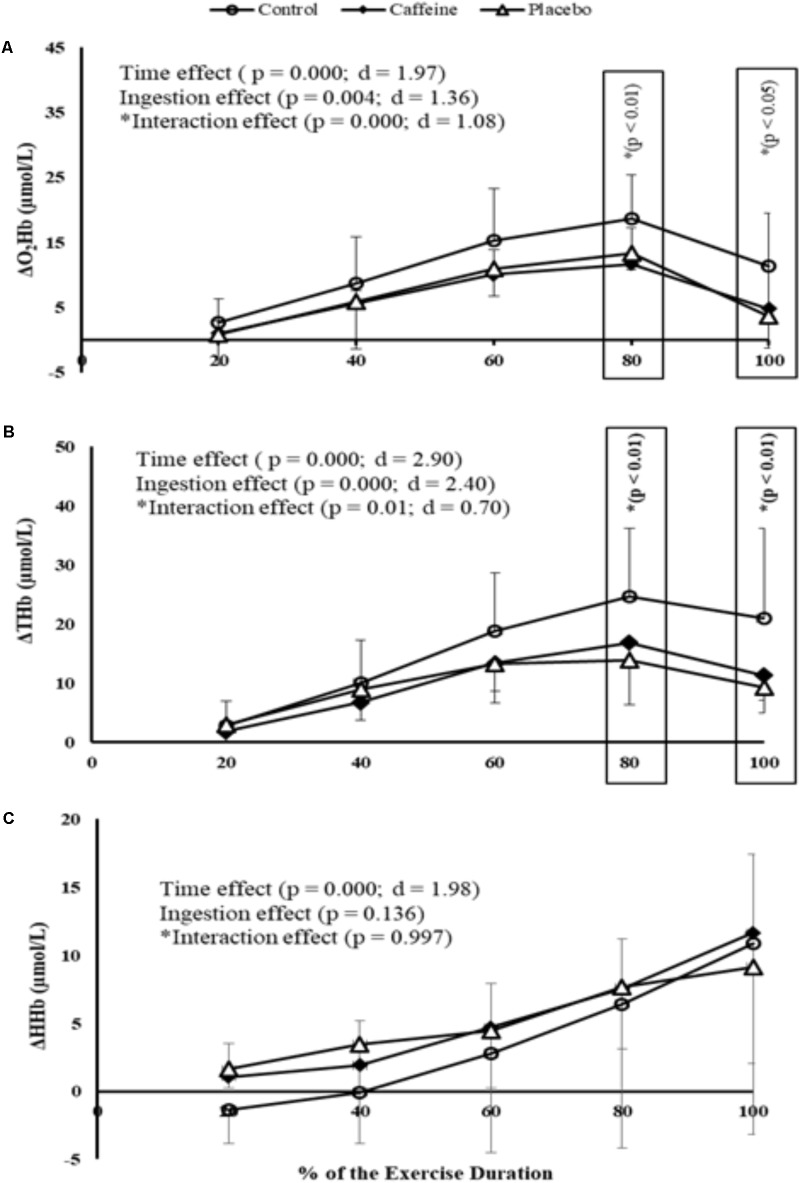
O_2_Hb **(A)**, THb **(B)**, and HHb **(C)** concentrations measured at prefrontal cortex (PFC) during MIT in control, caffeine and placebo (perceived as caffeine). Main effects of time and ingestion and time by ingestion interaction effects were reported in the figure. Values are mean (± SD).

Comparisons at relative intensities showed a time main effect in PFC oxygenation (*P* = 0.000; *F* = 15.55; *d* = 1.97) so that O_2_Hb concentrations increased up to 80% of the MIT duration and then decreased toward the exhaustion, regardless of the ingestion. An ingestion main effect was also observed (*P* = 0.004; *F* = 7.32; *d* = 1.36) because CAF (*P* = 0.002) and PLA (*P* = 0.05) elicited lower O_2_Hb concentrations (i.e., deoxygenation) than control during MIT. Additionally, a time by ingestion interaction effect was observed (*P* = 0.000; *F* = 4.68; *d* = 1.08) as O_2_Hb in both CAF and PLA was lower than control at 80% (*P* = 0.009 and *P* = 0.004, respectively) and 100% (*P* = 0.015 and *P* = 0.004, respectively) of the MIT duration. Accordingly, a time main effect (*P* = 0.000; *F* = 33.74; *d* = 2.90), an ingestion main effect (*P* = 0.000; *F* = 23.08; *d* = 2.40) and a time by ingestion interaction effect (*P* = 0.01; *F* = 1.96; *d* = 0.70) were detected in THb concentrations. Hence, overall responses were a lower THb in CAF (*P* = 0.000) and PLA (*P* = 0.000) than in control, mainly at 80% (*P* = 0.009 and *P* = 0.004, respectively) and 100% of the test duration (*P* = 0.001 and *P* = 0.000, respectively). Except for the time main effect (*P* = 0.000; *F* = 15.73; *d* = 1.98), neither ingestion main effect (*P* = 0.136) nor time by ingestion interaction effect (*P* = 0.997) was observed in HHb concentrations. Therefore, when compared to control overall responses were a deoxygenated PFC in CAF and CAF-perceived PLA, mainly from 80% of the MIT duration (**Figures 2A–C**).

**FIGURE 3:**
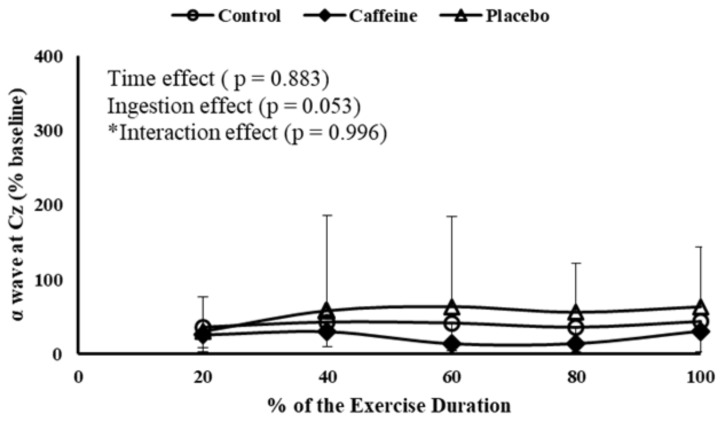
EEG α wave measured at primary motor cortex during MIT in control, caffeine and placebo (perceived as caffeine). Main effects of time and ingestion and time by ingestion interaction effects were reported in the figure. Values are mean (± SD).

Regarding MC activation, EEG α band at Cz position remained fairly steady throughout the MIT, as no time main effect was detected (*P* = 0.883). Accordingly, neither ingestion main effect (*P* = 0.053) nor time by ingestion interaction effect (*P* = 0.996) was observed in MC activation (**Figure 3**).

### Gaseous Exchange Responses

**FIGURE 4:**
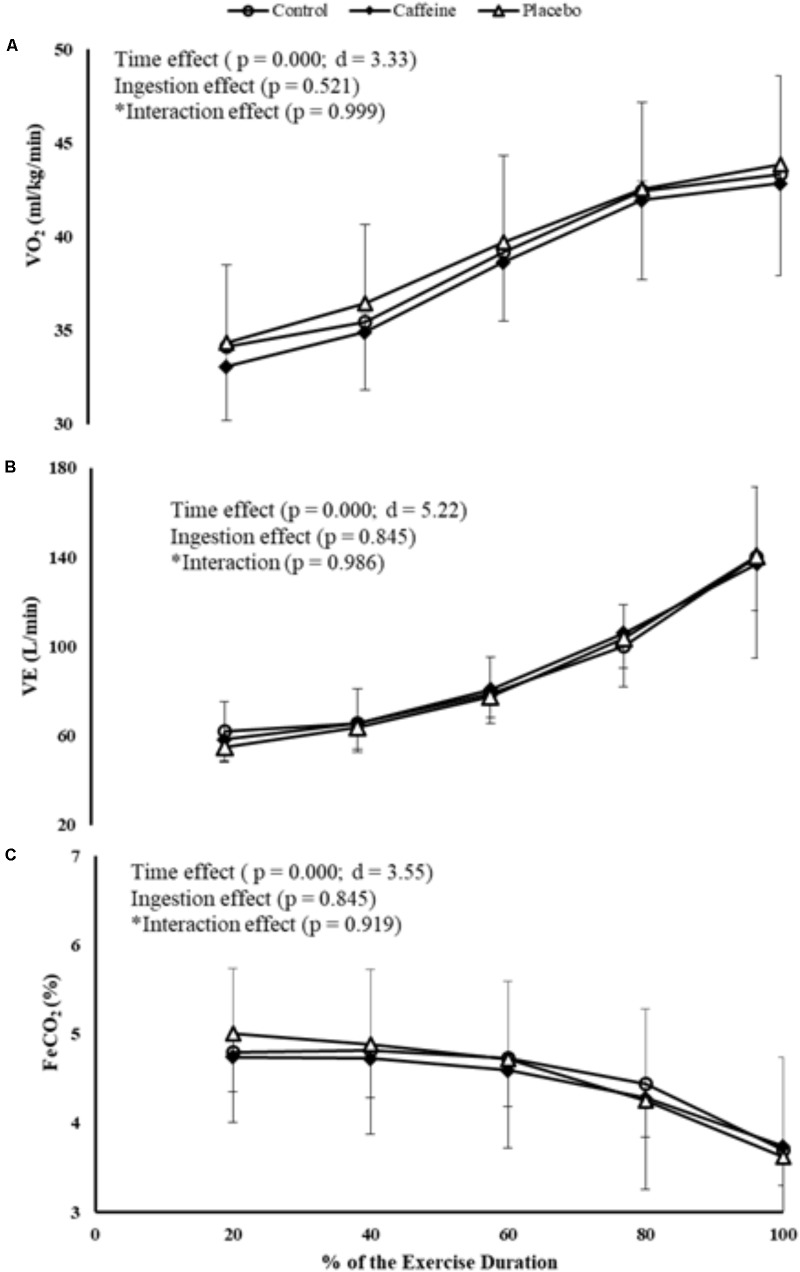
VO_2_ **(A)**, VE **(B),** and FeCO_2_ **(C)** responses during MIT in control, caffeine and placebo (perceived as caffeine). Main effects of time and ingestion and time by ingestion interaction effects were reported in the figure. Values are mean (± SD).

A time main effect was detected in VO_2_, regardless of the ingestion (*P* = 0.000; *F* = 44.39; *d* = 3.33), therefore VO_2_ increased progressively from 20% of the total MIT duration. Neither ingestion effect (*P* = 0.521) nor time by ingestion interaction effect (*P* = 0.999) was identified. Accordingly, VE increased progressively from 40% of the MIT duration regardless of the ingestion (*P* = 0.000; *F* = 109.21; *d* = 5.22), but neither ingestion main effect (*P* = 0.845) nor time by ingestion interaction effect (*P* = 0.986) was identified. In contrast, FeCO_2_ decreased from 80% of the total MIT duration (*P* = 0.000; *F* = 50.407; *d* = 3.55), but neither ingestion main effect (*P* = 0.845) nor time by ingestion interaction effect (*P* = 0.919) was identified (**Figures 4A–C**).

## Discussion

We investigated whether CAF and PLA perceived as CAF may improve motor performance during MIT, regardless of changes in MC activation and PFC deoxygenation. We found that both CAF and PLA improved MIT performance and increased PFC deoxygenation after 80% of the MIT, but had not effect on MC activation. As the effects of CAF and CAF-perceived PLA were comparable, our findings challenged the use of CAF to improve aerobic exercise performance and highlighted the potential PLA effect.

It has been proposed that CAF ingestion reduces cerebral perfusion (Nehlig et al., 1992) and PFC oxygenation (Yang et al., 2015). Accordingly, we found a CAF-induced hypoperfusion and PFC deoxygenation, as indicated by the lower ΔTHb and ΔO_2_Hb concentrations in CAF than in control at a mild absolute intensity (i.e., 150 W), respectively. Furthermore, the exercise-induced hypocapnia effect from 80% of the W_PEAK_ in MIT (Racinais et al., 2014) potentiated the PFC vasoconstriction and deoxygenation, as ΔTHb and ΔO_2_Hb concentrations were lower in CAF than in control from 80% of the MIT. Apparently, this was a combined effect of CAF and exercise-induced hypocapnia, rather than an isolated exercise-hypocapnia effect, as VE and FeCO_2_ responses were comparable between both conditions. Interestingly, our PLA design also reduced PFC oxygenation, as PFC ΔO_2_Hb concentration was lower in PLA than in control at a mild absolute intensity (i.e., 150 W). Moreover, interaction effects of the relative intensity comparisons indicated that the exercise-induced hypocapnia also potentiated the PLA-induced PFC deoxygenation, as both ΔO_2_Hb and ΔTHb concentrations were lower in PLA than in control from 80% of the MIT. Accordingly, PLA combined with the exercise-induced hypocapnia (rather than the isolated exercise-hypocapnia effect) induced to a PFC deoxygenation, given the comparable VE and FeCO_2_ responses between conditions. Taken together, these results suggest that the expectation of receiving CAF during PLA ingestion produced cerebral responses in the expected direction of the active substance (Wager et al., 2004, 2011; Liu, 2017), and that the exercise-induced hypocapnia from 80% of the MIT seemingly potentiated this response. Importantly, given the increases in EMG and W_PEAK_ after 80% of the TIM in CAF and CAF-perceived PLA conditions, these results also indicate that PFC deoxygenation limited neither muscle activation nor motor output, thereby showing that the PFC deoxygenation was a consequence of the ingested substances and exercise-induced hypocapnia.

Although the higher EMG and W_PEAK_ with CAF ingestion when compared to control, MC activation appeared to be similar, as suggested by unchanged EEG signal. Resembling CAF effects, MC activation remained unchanged throughout the MIT when participants ingested PLA perceived as CAF. The unchanged MC activation with CAF ingestion could be associated with a putative enhancement in self-sustained firing of motoneuron, as CAF has the ability to increase neuronal activity and excitability at spinal and supra-spinal levels (Walton et al., 2002; Kalmar, 2005). Likewise, our PLA intervention may also have enhanced the corticospinal excitability, as participants perceiving PLA as CAF may have boosted a cognitive top–down effect-induced corticospinal excitability (Pollo et al., 2008; Fiorio et al., 2014). Therefore, either as a result of the CAF ingestion or the expectation of receiving CAF, less excitatory input from MC seemed to be required to produce similar motor output during exercise (de Morree et al., 2014).

The lack of difference in cerebral and motor performance responses between CAF and PLA perceived as CAF during MIT challenges the pharmacological effects of CAF. Some studies have reported PLA-induced effects through different PLA settings (Benedetti et al., 2003; Fiorio et al., 2014), including cycling exercises (Foad et al., 2008). For example, improvements in cycling time trial performance were found when participants were given a PLA perceived as CAF, regardless of performance-related physiological measures such as VO_2_ responses (Foad et al., 2008). Accordingly, our PLA-deceived intervention induced motor performance improvements in the expected direction of the CAF ingestion, as previously reported (Foad et al., 2008; Saunders et al., 2016). Furthermore, our PLA design triggered an expectation-induced cerebral response similar to CAF, as suggested elsewhere (Wager et al., 2004, 2011; Liu, 2017). Together with previous results (Foad et al., 2008; Saunders et al., 2016), our findings question the use of CAF to potentiate motor performance and suggest that the expectation of ingesting CAF, rather than its pharmacological effects, has the ability to improve motor performance and change cerebral responses. Additionally, these results also challenged the classical view of a VO_2MAX_-limited exercise performance as we found improved MIT performance with CAF and CAF-perceived PLA, with no changes on VO_2_ and VE responses.

Results of the present study provide important insights regarding the use of double-blind designs in sports nutrition studies. Some suggested that double-blind designs are a possible source of bias in randomized trials (Kirsch and Weixel, 1988; Saunders et al., 2016), as performance outcomes on motor tests are influenced when participants identify the presence of a PLA intervention (Beedie et al., 2006; Foad et al., 2008). The use of a PLA-deceived design in the present study may have overestimated the PLA effects normally observed when participants are told they have a 50% chance of ingesting the actual active substance in a typical double-blind design (Vase et al., 2002). However, this design allowed quantifying the actual PLA effects on the aforementioned variables, as we were able to contrast CAF and PLA in a totally inert condition (i.e., baseline condition). In addition, comparisons at a mild absolute exercise intensity (i.e., 150 W) excluded metabolic-related confounding factors and confirmed the actual CAF and PLA effects on cerebral responses. Future nutrition studies should consider this aspect when investigating CAF effects on motor performance and cerebral responses to exercise.

### Future Perspectives

Our data provide new insight into the exercise regulation perspective. The PFC was suggested to play a role on exercise tolerance (Robertson and Marino, 2015a) as PFC is connected to premotor cortex areas, thereby regulating MC areas and motor output (Ridderinkhof et al., 2004; Loehrer et al., 2016) while integrating sensory afferents during exercise (Ekkekakis, 2009). Studies have shown that exercising at high intensities affects the PFC function and impairs cognitive performance when facing increases in interoceptive information (Ekkekakis, 2009; Mekari et al., 2015). Such a decline in PFC cognitive ability during high-intensity exercise is associated with the exercise hypocapnia-induced PFC deoxygenation so that an impaired PFC cognitive ability from 80% of the TIM was expected to be potentiated by CAF and PLA ingestion. However, CAF and PLA maintained the ability to increase motor output throughout the MIT, regardless of the greater PFC deoxygenation after 80% of the MIT. A recent study suggested that the capacity to perform maximal aerobic exercise may be related to tolerating PFC deoxygenation while not impairing MC activation and motor output (Pires et al., 2016). From a cerebral regulation perspective, it might be worth verifying CAF and PLA effects on different maximal aerobic exercise modes such as a cycling time trial, as PFC deoxygenation and MC activation may depend on the exercise mode under consideration (Pires et al., 2016).

### Methodological Aspects and Limitations

The use of EEG technique to monitor changes in cortical activation during exercise has been criticized, as artifacts derived from the upper body movements can introduce motion artifacts in the EEG data (Thompson et al., 2008). During the experimental setup, we used active electrodes, fixed cables and electrodes, and familiarized participants to maintain eyes opened and upper limbs as steady as possible during EEG measures.

The use of NIRS technology to monitor cerebral responses to exercise may also have some limitations as this technique assesses superficial, rather than deep tissues (Ekkekakis, 2009). Therefore, our inferences were limited to the specific sites measured in the present study and cannot be extrapolated to subcortical structures. Moreover, as regional blood flow responses to cortical activation typically exceeds the metabolic demand, how much of the O_2_Hb reduction negatively impacted PFC function is currently unknown. Nevertheless, NIRS is a non-invasive technology that monitors tissue oxygenation with a good temporal resolution and reasonable signal-to-noise ratio, thereby enabling the access to important cerebral responses to exercise. Additionally, it is important to highlight that a cause-effect relationship between PFC and MC should be interpreted with caution, as PFC is likely connected with premotor cortex areas (Loehrer et al., 2016) so that a direct connection to MC areas are still under scrutiny (Ridderinkhof et al., 2004).

## Conclusion

In summary, results of the present study showed that both CAF and CAF-perceived PLA improved MIT performance, despite unchanged MC activation and greater PFC deoxygenation from 80% of the MIT. These results challenged the effectiveness of CAF as ergogenic to potentiate motor performance in maximal exercises.

## Author Contributions

FOP, TN, ASCG, and CU: contributed to this study, conceiving and designing the experiments. FOP, CdA, EF, FM, and RC: collecting and analyzing the data. FOP, EF, and CU: writing the manuscript. CdA, RC, FM, TN, ASCG, and CU: criticizing and reviewing the manuscript. All the listed authors approved the final version of the manuscript. This study was performed at the Clinics Hospital, University of Campinas, Brazil.

## Conflict of Interest Statement

The authors declare that the research was conducted in the absence of any commercial or financial relationships that could be construed as a potential conflict of interest.

## Footnotes

1 http://www.nmr.mgh.harvard.edu/PMI/resources/homer/home.htm
